# Urban Leptospiral Meningoencephalitis Associated With Bottle Recycling: A Case Report and Literature Review

**DOI:** 10.7759/cureus.110404

**Published:** 2026-06-07

**Authors:** Abeer Qasim, Saran Lal Ajai Mokan Dasan, Venkata Sri Ramani Peesapati, Abhishrut P Jog, Hammad Ashraf, Misbahuddin Khaja

**Affiliations:** 1 Internal Medicine, BronxCare Health System, New York, USA

**Keywords:** bottle recycling, leptospirosis, meningitis, public health concern, rodent exposure

## Abstract

Leptospirosis is a preventable zoonotic infection that can present with a broad spectrum of clinical features, ranging from mild flu-like illness to severe multi-organ dysfunction. Meningitis caused by *Leptospira* is rare in developed countries and among the urban population. We describe the case of a 50-year-old male with schizophrenia and hypothyroidism who presented with seizures, jaundice, shock, and respiratory failure. Initial evaluation revealed severe metabolic acidosis, acute kidney injury, transaminitis, and thrombocytopenia. Lumbar puncture showed xanthochromic cerebrospinal fluid with lymphocytic predominance. Serum polymerase chain reaction confirmed leptospiral exposure. The patient was treated with antibiotics. He recovered following a complicated and prolonged hospital course. Epidemiologic history revealed daily collection of discarded bottles in areas with reported rodent exposure. Our patient’s occupational exposure from bottle collection likely facilitated *Leptospira* infection. The urinary pneumococcal antigen detection during admission led to anchoring bias and subsequent delayed recognition of leptospiral meningitis. This case highlights the importance of a thorough exposure history and broad diagnostic testing in atypical presentations of meningitis, particularly when occupational or environmental risk factors are present.

## Introduction

Leptospirosis is a worldwide zoonotic disease that affects both humans and animals. Direct contact with infected animals or their urine is a common route of transmission, making occupations involving animals or exposure to animal waste a significant risk factor. Leptospires can survive for days to months in urine-contaminated soil and freshwater [1]. Clinical manifestations range from asymptomatic or mild self-limited symptoms to severe, potentially fatal complications such as Weil’s disease and pulmonary hemorrhage syndrome. The severe form of the disease can affect multiple organ systems, leading to complications such as myocarditis, acute renal failure, rhabdomyolysis, aseptic meningitis, and acute liver failure, with resultant multi-organ failure and even death [2,3]. Efforts to control leptospirosis include active surveillance, early detection of the epidemiological source, and targeted interventions. Improving sanitation, implementing rodent-control measures, and promoting public awareness of the disease and preventive measures are crucial, as there are limited advances in vaccine development owing to the presence of 200 disease-causing serovars. Here, we present the case of a 50-year-old male who exhibited severe leptospirosis with a rather unique occupational exposure.

This article was uploaded as a preprint under the title, “A penny for your CSF - an unexpected consequence of bottle recycling: meningitis due to Leptospira and Streptococcus - a case report” on Research Square on February 16, 2026.

## Case presentation

A 50-year-old male with a past medical history of schizophrenia and hypothyroidism from the Bronx, New York, was brought to the emergency department following a witnessed seizure at home. Collateral history from his mother indicated that, two to three days before presentation, he had developed body aches, nausea, vomiting, loss of appetite, and inability to tolerate oral intake. The patient was lethargic on arrival and was unable to provide pertinent history.

On arrival, he was hemodynamically unstable, with a blood pressure of 75/50 mmHg, temperature of 94.4°F, heart rate of 140 beats/minute, respiratory rate of 40 breaths/minute, and oxygen saturation of 74% on room air. Physical examination revealed jaundice with icteric sclera, lethargy, and absence of meningeal signs, although he responded to questions and commands. He was intubated for acute respiratory failure in the context of suspected septic shock and was admitted to the intensive care unit. Table 1 summarizes the laboratory results at presentation. Routine hematology and chemistry tests were performed in the hospital clinical laboratory using automated analyzers per institutional protocols.

**Table 1:** Laboratory investigations (blood and urine). AST: aspartate aminotransferase; ALT: alanine aminotransferase; ALP: alkaline phosphatase; GGT: gamma-glutamyl transferase; INR: international normalized ratio; TSH: thyroid-stimulating hormone; RBC: red blood cells; WBC: white blood cells; HPF: high-powered field

Test	Result	Reference range
Hemoglobin	10.7 g/dL	Male: 13.5–17.5 g/dL
White cell count	20.1 × 10^3^/mL	4–11 × 10^3^/mL
Platelet count	63 × 10^3^/mL	150–450 × 10^3^/mL
Creatinine	1.8 mg/dL	Male: 0.74–1.35 mg/dL
Lactic acid	14.5 mmol/L	0.5–2.2 mmol/L
AST	320 U/L	10–40 U/L
ALT	152 U/L	7–56 U/L
ALP	135 U/L	44–147 U/L
Total bilirubin	13.8 mg/dL	0.1–1.2 mg/dL
Direct bilirubin	4.3 mg/dL	0.0–0.3 mg/dL
GGT	38 U/L	9–48 U/L
Ammonia	180 µmol/L	15–45 µmol/L
Bicarbonate	5 mEq/L	22–29 mEq/L
Anion gap	48 mEq/L	8–12 mEq/L
INR	1.09	0.8–1.1
Free thyroxine	0.66 ng/dL	0.8–1.8 ng/dL
T3	26.9 ng/dL	80–200 ng/dL
TSH	31.3 mIU/L	0.4–4.5 mIU/L
Creatinine kinase	4,106 U/L	Male: 52–336 U/L
Urine drug screen	Positive for cannabinoids	Negative
Urine analysis		
Proteinuria	Turbid proteinuria	Clear
Blood	300+	Negative
Leukocyte esterase	500+	Negative
WBC	Few WBCs	0–5/HPF
RBC	Few RBCs	0–2/HPF
Bilirubin	Moderate	Negative
Arterial blood gas		
pH	7.18	7.35–7.45
PaCO_2_	18 mmHg	35–45 mmHg
PaO_2_	68 mmHg	80–100 mmHg
Bicarbonate	7 mEq/L	22–26 mEq/L
Lactate	14.5 mmol/L	0.5–2.2 mmol/L

Chest X-ray, as well as computed tomography (CT) scans of the head, cervical spine, abdomen, and pelvis, revealed no significant abnormalities. The patient was started on broad-spectrum antibiotics (ceftriaxone 2 g twice daily, doxycycline 100 mg twice daily, and vancomycin by level) at presentation. Subsequently, hemodialysis was initiated due to high anion gap metabolic acidosis and worsening acute kidney injury. Persistent seizures prompted the addition of anti-epileptics (levetiracetam) and lumbar puncture on hospital day two (Table 2). Initial cerebrospinal fluid (CSF) analysis showed elevated protein, modestly elevated opening pressure, xanthochromia, and increased white blood cell count, suggestive of inflammatory meningoencephalitis. Infectious workup revealed a positive urine pneumococcal antigen result by rapid immunochromatographic (lateral flow) assay, indicating possible pneumococcal infection/exposure. This led to an early, premature diagnosis of possible bacterial meningitis. The negative bacterial culture and persistent neurological symptoms prompted blood leptospiral polymerase chain reaction (PCR) (*Leptospira* DNA, qualitative, real-time PCR) and repeat CSF analysis (Table 3).

**Table 2:** Cerebrospinal fluid studies. WBC: white blood cells; RBC: red blood cells; LDH: lactate dehydrogenase

	On day 2 of admission	On day 14 of admission	Reference range
Opening pressure	22 cmH_2_O	15 cmH_2_O	5–20 cmH_2_O
Cell count and differential			
Color	Xanthochromia	Xanthochromia	-
Appearance	Clear	Cloudy	-
WBC	17 cells/mL	10 cells/mL	0–5 cells/mL
RBC	825 cells/mL	3,150 cells/mL	0 cells/mL
Segmented count	69%	19%	-
Lymphocyte count	28%	78%	-
Monocyte	2%	2%	-
Eosinophil	1%	1%	-
Macrophage	Few	None	-
Glucose	90 mg/dL	42 mg/dL	50–80 mg/dL
Protein	269 mg/dL	93 mg/dL	15–45 mg/dL
LDH	126 U/L	-	<40 U/L
Aerobic culture	No growth of organisms	No growth of organisms	-

**Table 3:** Microbiologic and infectious disease testing.

Tests	Results	Expected result
Urine *Legionella* antigen	Not detected	Not detected/Negative
Urine pneumococcal antigen	Positive	Not detected/Negative
Urine culture	No growth	No growth/Negative
Cryptococcal antigen	Negative	Not detected/Negative
Blood culture	*Staphylococcus hominis*, *Corynebacterium* species (likely contaminants, as subsequent blood cultures were negative twice)	No growth/Negative
Fungal blood culture	Negative	No growth/Negative

Twelve days later, *Leptospira* PCR/DNA was detected in the patient’s blood. Repeat lumbar puncture was also performed for persistent neurological symptoms. Leptospira PCR was not performed on the CSF sample due to institutional laboratory limitations. With positive leptospiral PCR and CSF findings, the patient was diagnosed with leptospiral meningoencephalitis. Antibiotics were switched in view of renal and hepatic impairment to intravenous penicillin G 2 million units every four hours for a total duration of 10 days.  Subsequently, the patient underwent a tracheostomy and percutaneous endoscopic gastrostomy placement due to delayed neurological recovery. The patient no longer required hemodialysis after four weeks of adequate renal recovery. He was discharged to a nursing home for short-term rehabilitation.  Since discharge, the patient has undergone reversal of the tracheostomy and removal of the percutaneous endoscopic gastrotomy. He later disclosed that he was dwelling in the streets and was regularly roaming the streets to collect bottles from trash cans for money, without any protective gear, an activity that brought him into frequent contact with rodents and rodent droppings. At follow-up, he was doing well, attending outpatient visits, and had resumed his usual daily activities, including bottle recycling.

## Discussion

The term “leptospirosis” was first used in 1886, and the disease was named “leptospirosis icterohemorrhagica,” referring to its ability to cause icterus and scleral hemorrhage [4]. Leptospirosis is a neglected tropical disease caused by the spirochetal bacteria belonging to the genus Leptospira, with an estimated 1 million human cases occurring annually worldwide, including nearly 60,000 deaths [5,6]. In the United States, the incidence of leptospirosis is 100-150 cases annually [7]. The disease is reportable in all US states. Ten states in the US have container deposit legislation, popularly known as “bottle bills.” In New York, the Returnable Container Act (RCA) has been in effect since July 1, 1983, and reverse vending machines are operational nationwide for this purpose. The return of empty containers to these machines results in payment of 5 cents per container [8].

Our patient used to collect empty bottles from trash cans and streets, and was earning money by returning them to a reverse vending machine. He also encountered rodents daily when handling the trash cans. Inoculation likely happened due to exposure without any protective gear.  In 2024, New York City issued a health advisory stating that between 2001 and 2023, the city saw 98 cases of leptospirosis. Overall, 94% of the cases were seen in males, with a median age of 50 years. Most of the cases were found in the Bronx [9].

Leptospirosis has a global presence in nearly all tropical and temperate regions and is one of the most widespread zoonotic infections worldwide [3]. It was estimated that 500,000 cases of severe leptospirosis were diagnosed worldwide each year [6]. These infections occur when individuals encounter environmental sources such as animal urine, contaminated water or soil, or infected animal tissue [10,11]. *Leptospira* species can infect a wide range of animals, including mammals such as rats [12]. *Leptospira* enter the body through openings in the skin, such as lesions or abrasions, as well as through the lining of the mouth or waterlogged skin after prolonged immersion [7]. Various risk factors for leptospirosis are listed in Table 4 [13,14].

**Table 4:** Risk factors for leptospirosis.

Types of risks	Associated examples
Occupational	Farmers and ranchers, veterinarians, loggers, sewage workers, rice field workers, military personnel
Recreational	Freshwater swimming, canoeing and kayaking, trail biking, hunting, walking in the farmland
Household	Pet dogs, domesticated livestock, rainwater catchment systems, rodent infestation

The clinical symptoms of leptospirosis resemble those of infections such as dengue or influenza [15].* *Severe form of the disease, like meningitis, is rare and was observed only in 5% of cases [16]. If left untreated, leptospirosis meningitis can progress to more severe complications, including coma, meningoencephalitis, hemiplegia, transverse myelitis, or Guillain-Barré syndrome [15].  After the acute or septicemic phase, which typically lasts about one week, the immune phase begins, characterized by antibody production [17]. However, jaundice takes longer to clear [18].

Severe leptospirosis is also marked by renal and hepatic dysfunction. Leptospirosis can present in two distinct clinical syndromes: anicteric and icteric [19]. Hemorrhagic pulmonitis is another severe form of the disease with higher mortality rates. Rarer manifestations include purely neurological manifestations, which may present as meningitis/meningoencephalitis [11,20].

Various methods for diagnosing the organism directly or detecting its components, such as culture, microscopy, and molecular techniques, are available, alongside serological tests such as the microagglutination test (MAT), enzyme-linked immunosorbent assay (ELISA), and rapid diagnostic tests (RDTs). The gold standard for diagnosis is culture of clinical specimens (urine, blood, or CSF) or MAT, which is considered the reference test [21]. The role of PCR in detecting leptospiral DNA is well reported. PCR-based DNA testing is highly specific when compared to antibody-based techniques. A study conducted in a Baltimore STI clinic for reasons unrelated to leptospirosis showed 16% positivity for leptospiral antibody [22]. In addition, PCR can detect leptospiral DNA in CSF even during the acute phase. In contrast, only 4% and 9% patients showed positive results for ELISA-IgM and MAT during the acute phase [23]. Therefore, PCR offers benefits in promptly diagnosing leptospiral meningitis, especially when antibodies are either absent or present in low levels in CSF or blood [24]. This has been corroborated in a flood-related outbreak in Sri Lanka, when the quantitative PCR assay was shown to be more sensitive than other serological tests [25].

Treatment guidelines for leptospirosis recommend penicillin, cephalosporins, doxycycline, and chloramphenicol, depending on the severity of illness [26]. Leptospira exhibits susceptibility to a wide range of antibiotics. For severe disease, intravenous penicillin had been the drug of choice for decades [27]. Initially, our patient received vancomycin, ceftriaxone, and doxycycline for unclear sepsis. Leptospiral DNA was detected in the patient’s blood on day 12 of admission, leading to a diagnosis of leptospiral meningoencephalitis, and antibiotics were switched to penicillin G in view of renal and hepatic dysfunction.

The use of steroids in handling severe complications caused by leptospirosis is a matter of debate and lacks sufficient evidence [28]. Supportive care includes managing symptoms such as fever, pain, and dehydration. Patients with severe cases may require hospitalization. Despite optimal treatment, the mortality rate remains elevated in cases of severe leptospirosis [29]. The case fatality rate (CFR) was reported to be 15% in some studies [18]. The CFR was reported at 6.7% when presenting with hepatic or renal involvement, with mortality rising as high as 50-70% when presenting with hemorrhagic pneumonitis [18]. Other predictors with a high mortality rate include age ≥70 years, breathlessness, lung involvement, oliguria, use of vasopressors, administration of steroids, the need for ventilator support, and blood transfusion [30].

Although different animal species could be the primary setting in different epidemiological sources, the basic principles of prevention include source reduction, environmental sanitation, and the use of safety gear when handling waste with possible rodent contamination. Cattle immunization was attempted in 1992 but was later abandoned as it could not eradicate infection. Chemoprophylaxis in travellers to endemic regions was almost 100% effective. Attempts at vaccine development are still ongoing. Previously developed vaccines had limited effectiveness owing to the large number of serovars and the bacterial ability to evade immunity [18].

## Conclusions

We present a severe case of leptospirosis with meningoencephalitis, circulatory shock, and acute kidney injury in a patient with repeated environmental exposure to rodent exposure during bottle recycling. Although leptospirosis is often associated with tropical or rural exposures, this case emphasizes that urban rodent exposure remains an important and underrecognized risk factor, particularly in high-risk settings such as trash handling and bottle collection. The positive urinary pneumococcal antigen created diagnostic uncertainty and may have contributed to anchoring bias, emphasizing the need to interpret non-specific or indirect microbiologic results cautiously when the clinical picture remains unexplained. In patients presenting with aseptic meningitis or meningoencephalitis with jaundice, renal dysfunction, and shock, clinicians should maintain a high index of suspicion for leptospirosis and pursue early molecular or serologic testing. Early recognition, appropriate antimicrobial therapy, supportive organ replacement, and careful exposure history assessment are critical to improving outcomes and identifying preventable public health risks in urban communities.
